# Neurotrophins: Neuroimmune Interactions in Human Atopic Diseases

**DOI:** 10.3390/ijms24076105

**Published:** 2023-03-24

**Authors:** Tobias Weihrauch, Maren M. Limberg, Natalie Gray, Martin Schmelz, Ulrike Raap

**Affiliations:** 1 Division of Experimental Allergy and Immunodermatology, Faculty of Medicine and Health Sciences, University of Oldenburg, 26129 Oldenburg, Germany; 2 Department of Experimental Pain Research, MCTN, Medical Faculty Mannheim, University of Heidelberg, 68167 Mannheim, Germany; 3 University Clinic of Dermatology and Allergy, University of Oldenburg, 26133 Oldenburg, Germany

**Keywords:** neurotrophin, pruritus, atopic dermatitis, allergic rhinitis, allergic asthma

## Abstract

Allergic diseases are accompanied by a variety of symptoms such as pruritus, coughing, sneezing, and watery eyes, which can result in severe physiological and even psychological impairments. The exact mechanisms of these conditions are not yet completely understood. However, recent studies demonstrated a high relevance of neurotrophins in allergic inflammation, as they induce cytokine release, mediate interaction between immune cells and neurons, and exhibit different expression levels in health and disease. In this review, we aim to give an overview of the current state of knowledge concerning the role of neurotrophins in atopic disorders such as atopic dermatitis, allergic asthma, and allergic rhinitis.

## 1. Introduction

Atopic diseases are associated with symptoms such as skin inflammation, pruritus, cough, dyspnea, sneezing, or watery eyes, which can strongly impair patients’ quality of life. The exact mechanisms involved in inflammation and the development of these symptoms are not yet fully understood. However, recent studies revealed that immune cells are capable of interacting with neurons, therefore indicating neuroimmune interactions in which neurotrophins play an important role. Here, we want to examine the role of neurotrophins in neuroimmune interactions more closely, and summarize the state of knowledge on their contribution to inflammation in allergic diseases such as pruritic skin diseases like atopic dermatitis (AD) but also allergic rhinitis (AR), asthma, and rhinoconjunctivitis (ARC).

The family of neurotrophins consists of four identified members: brain-derived neurotrophic factor (BDNF), nerve growth factor (NGF), neurotrophin 3 (NT-3), and neurotrophin 4/5 (NT-4/5). They bind to tropomyosin-related kinase (Trk) receptors. NGF has a high affinity for TrkA, while BDNF and NT-4/5 preferentially bind to TrkB, and NT-3 to TrkC but also TrkA and TrkB. Further, all neurotrophins bind to the pan-neurotrophin receptor (p75NTR), a member of the TNFR/Fas/CD40 superfamily, with low affinity [1]. A variety of immune cells, such as T cells [2,3,4], B cells [3,5], mast cells [6], eosinophils [7], and macrophages [8,9], are capable of releasing neurotrophins (Table 1). The neurotrophins possess a common structure and overlap their functions. Each consists of a signal sequence and a prodomain attached to the actual neurotrophin sequence. This suggests that a post-transcriptional step is required to form the mature proteins and to specify their biological actions. The first receptor to be identified was p75NTR, initially reported as a receptor for NGF but later found to also bind all other neurotrophins. The receptor is generally known to regulate survival, apoptosis, neurite outgrowth, and migration after activation. In contrast, the specific Trk receptors mediate distinct biological actions via neurotrophin binding, leading to dimerization and autophosphorylation. Trk receptors and p75NTR can not only be activated separately but also be coactivated. The partially opposing biological effects after Trk receptor and p75NTR activation can be explained by the preferential binding of the two receptor types to mature and proneurotrophins, respectively [10].

## 2. Brain-Derived Neurotrophic Factor

**Figure 1:**
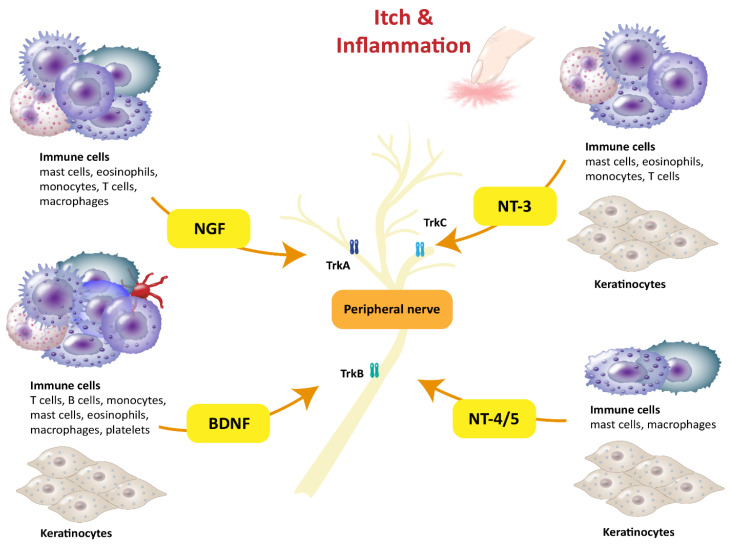
Immune cells release neurotrophins that affect peripheral nerves in the skin.

BDNF was named after it was first extracted from porcine brain tissue and identified as a survival factor for neuronal populations that are not responsive to NGF [10]. Beyond its role in neurons, BDNF is also released by keratinocytes, melanocytes, fibroblasts, endothelial cells [37], platelets [15], and several immune cells such as T cells, B cells, monocytes [3], macrophages [11], mast cells [12], and eosinophils [7] (Table 1, Figure 1). Out of all these immune cells, the effect of BDNF release is best characterized in eosinophils.

### 2.1. Atopic Dermatitis and BDNF

**Figure 2:**
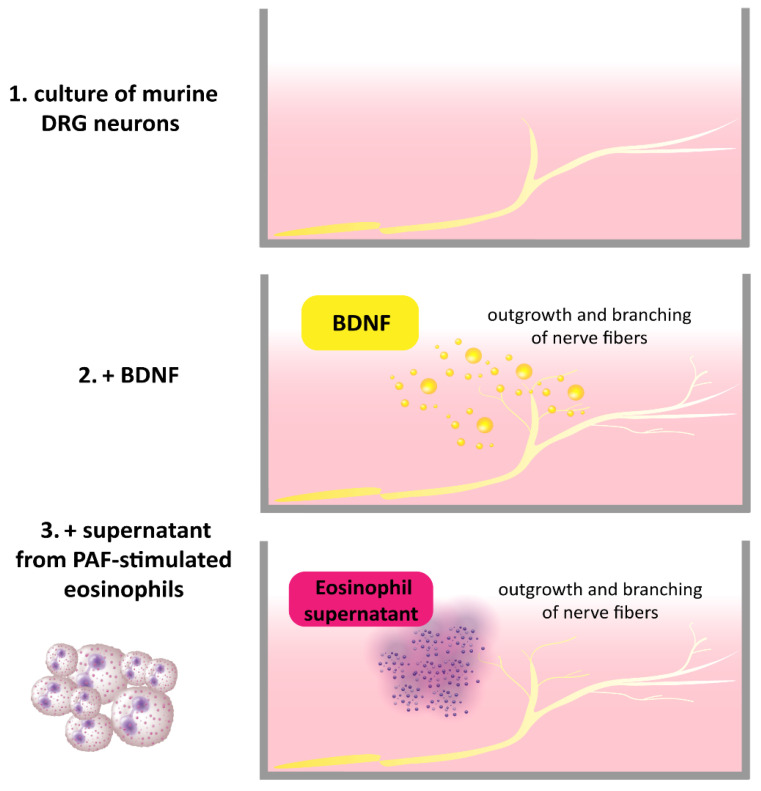
BDNF released by PAF-stimulated AD eosinophils induces the outgrowth and branching of mouse DRG neurons [7].

Previously, it was shown that BDNF-positive eosinophils were located in close vicinity to βIII-Tubulin positive nerve fibers in the skin of patients with AD [7]. Further, the number of eosinophils found in close proximity to dermal nerve fibers was increased in AD, and BDNF levels in neurons were also elevated. Stimulation of murine dorsal root ganglia (DRG) with BDNF causes outgrowth and branching of nerve fibers [7]. In this regard, we showed that eosinophils from AD patients expressed functional BDNF by inducing its release with platelet-activating factor (PAF) and subsequently treating DRG neurons with the eosinophil supernatants. The DRG neurons responded with an outgrowth of neurites, demonstrating the ability of eosinophils to structurally modulate sensory nerves [7] (Figure 2). The modulating effect of other immune cells through BDNF on nerve fibers has not yet been described, although a similar effect to that of eosinophils is conceivable. Mast cells are known to be localized in the vicinity of peripheral nerve endings in AD [44], which suggests that neuroimmune interactions might also occur with BDNF. Furthermore, morphological analyses of cutaneous nerves in skin biopsies of AD patients revealed increased numbers of axons in the upper dermis and a significantly higher density of axons in comparison with control subjects [7]. The numbers and length of axons in AD were also higher in lesional skin than in non-lesional skin [28]. This causes a higher density of nerve fibers in the epidermis of AD patients in comparison to control subjects. The increased density is suggested to be associated with functional impairments and involvement in itch sensation. This may be due to hyperinnervation of the skin, which might increase the responsiveness to exogenous trigger factors and endogenous pruritogens [45,46,47]. However, this hypothesis remains controversial since the nerve fiber density does not necessarily correlate with scratching behavior, as shown in a mouse model [48].

Furthermore, BDNF might also play a pivotal role in AD, as the plasma, serum, and eosinophils of AD patients contain higher levels of BDNF than those measured in healthy controls [13]. Interestingly, serum levels were also found to correlate with disease severity in adults [31] and children with AD, as well as with scratching behavior [49]. Moreover, increased BNDF levels in AD correlate with the amount of released eosinophil cationic protein (ECP) [49]. Analysis of skin biopsies from AD patients also revealed that eosinophil peroxidase was increased in lesional skin compared to non-lesional and healthy skin [28]. This is interesting, seeing as BDNF has been reported to induce the release of eosinophil peroxidase in eosinophils [24]. However, not only higher expression levels but also findings about the effect of BDNF on eosinophil function provide better insights into its role in AD. It has been shown that BDNF inhibits the apoptosis of eosinophils [13], which in turn might lead to prolonged and amplified total cytokine release and thus could potentially enhance inflammation. Furthermore, BDNF induces chemotactic migration of eosinophils in AD, opposing the effect observed in nonatopic patients [13], which is likely to support eosinophil recruitment into inflamed tissue. However, not only BDNF but also its receptor TrkB, which is expressed in T cells [50], B cells [51], and eosinophils [39] (Figure 1), and the low-affinity receptor p75NTR are suggested to contribute to the pathogenesis of AD. Both receptors exhibit higher expression levels in AD than in healthy control subjects [13]. Upregulation of TrkB was also observed in human peripheral blood eosinophils from AD patients [39]. These findings indicate that BDNF is a pivotal player in AD, as it was shown that eosinophils release biologically active BDNF in close vicinity to nerve fibers, modifying their morphology.

### 2.2. BDNF in Allergic Rhinitis

BDNF has not only been identified as a crucial factor in skin inflammation like AD but also in AR, which was observed in a study with AR and healthy patients. The expression of BDNF in the nasal mucosa of patients with AR after provocation with allergens was increased when compared to non-allergic subjects [14]. This increase was also positively correlated with the total nasal symptom score in AR after nasal provocation. In contrast, no increased BDNF levels could be measured in the bronchial mucosa after allergen-provocation, though its expression in AR was higher than in healthy patients. The study demonstrated that BDNF serum levels tended to be increased in AR, and allergen provocation further elevated BDNF levels in serum after 24 h of incubation [14]. Interestingly, the BDNF receptor TrkB was downregulated in mast cells after nasal provocation, though patients with AR still exhibited higher expression levels than healthy controls [14]. Levels of BDNF and its receptor might be influenced by other cytokines; however, variants of the BDNF gene have also been identified to have an impact on BDNF release. A single nucleotide polymorphism was found to alter protein function, including the secretion process of BDNF, and was also associated with an increased risk of moderate and severe AR [52]. Besides AR, these genetic variations have also been identified to be associated with a higher susceptibility to allergic asthma [53]. The effect of BDNF on nerve fibers in AR has not been described; however, patients with allergic rhinitis have a greater number of vasoactive intestinal polypeptide (VIP)-positive nerve fibers in the mucosal tissue of the nasal conchae than control subjects. VIP is known for causing vasodilatation and glandular secretion [54]. This suggests higher sensitivity to environmental allergens and more severe symptoms such as sneezing or nasal obstruction. Involvement of BDNF in the morphological changes in AR as observed in the skin seems possible.

### 2.3. BDNF in Allergic Asthma

Contrary to the findings in AD, the topic of increased BDNF serum levels in asthma is controversial. While a study from Lommatzsch et al. demonstrated significantly higher serum and plasma levels of BDNF in patients with allergic asthma [15], Joachim et al. did not observe increased serum levels of BDNF in allergic asthma when compared to healthy control subjects [16]. However, BDNF levels in the serum of asthmatic children were found to be inversely correlated with the expiratory volume [42]. Additionally, it has also been observed that BDNF levels in bronchoalveolar lavage fluid (BALF) of asthma patients were elevated after provocation with allergens [17]. These findings might indicate that the release of BDNF in acutely inflamed airways occurs in a more local manner without necessarily increasing systemic levels. BDNF has been described to be expressed by both the bronchial and alveolar epithelium, fibroblasts, the vascular epithelium, and airway smooth muscle cells in human lung tissue [55]. BDNF, derived from human airway smooth muscle cells, has been shown to regulate the release of calcium from the sarcoplasmic reticulum in response to agonists via TrkB. This is suggested to have an impact on the contractility of airway smooth muscle cells in asthma [18]. Another source of high amounts of BDNF are platelets, which were shown to contain reduced amounts of BDNF during respiratory tract infections. This might be due to the enhanced release of BDNF during inflammation [56]. Furthermore, elevated concentrations of BDNF in platelets correlate with bronchial hyperresponsiveness in allergic asthma, and inhibition of BDNF reduces neuronal hyperreactivity to allergens, as shown in an animal model [15]. As observed in the skin, BDNF might change the morphology of nerves in the airways since nerve fiber density was described to be higher in eosinophilic asthma, which was associated with a higher sensitivity to environmental stimuli. It was shown in mice that eosinophils increase epithelial innervation and neuronal reflex bronchoconstriction [57]. Since eosinophils in the skin cause an outgrowth of nerve fibers via BDNF, it might have the same effect on nerves in the airways. All these findings imply that BDNF is also involved in atopic diseases of the respiratory system.

In summary, differing expression levels of BDNF in serum and local fluids of patients with AD and symptom-free subjects indicate that BDNF plays an important role in allergic diseases. This is further substantiated by the fact that allergen provocation also induces varying BDNF levels in AR and allergic asthma. Furthermore, BDNF also promotes morphological changes in neurons in AD as a result of neuroimmune interactions.

## 3. Nerve Growth Factor

Of all four neurotrophins, NGF was the first to be identified by Rita Levi-Montalcini in the early 1950s and is the best characterized [58]. It was first described as a nerve growth stimulating factor and discovered to be a diffusible substance found in cell-free homogenates from sarcomas that induces neurite outgrowth in sympathetic and sensory ganglia explants [59]. However, NGF not only plays a role in the nervous system but also modulates the immune system, as a variety of immune cells such as mast cells [19], basophils [20], eosinophils [21], monocytes [22], and lymphocytes [2,60] are responsive to NGF (Table 1, Figure 1).

### 3.1. Skin Inflammation and NGF

In eosinophils, stimulation with NGF inhibits apoptosis [23] and increases expression of IL-4 [24], whereas in basophils, NGF induces the secretion of IL-13, which is elevated in allergic subjects [25]. IL-4 facilitates the release of further IL-4 and other AD-related proinflammatory cytokines such as IL-5, IL-6, and IL-13 [61]. Furthermore, IL-4 induces the production of immunoglobulin E (IgE) by B cells [61] and activates sensory neurons, which also occur in response to IL-13 [62]. IL-13 also promotes production of the pro-Th2 cytokine thymic stromal lymphopoietin (TSLP) in keratinocytes, which in turn increases levels of IL-4 and IL-13 [61]. Thus, NGF enhances inflammation by starting a cascade of mediator release through eosinophils and basophils, which in turn can recruit other immune cells.

NGF is further known to cause an upregulation of the neuropeptides substance P (SP) and calcitonin gene-related peptide (CGRP) [26]. SP is released by sensory nerves in the skin and is known to be involved in itch signaling through the neurokinin 1 receptor (NK_1_R). It promotes skin inflammation by inducing mast cell degranulation and also by causing the release of NGF from keratinocytes [63]. Both SP and CGRP are suggested to promote neuronal sensitization [26]. Besides cytokine release and changes in the sensitivity of neurons, NGF also affects the recruitment of immune cells to nerves in inflamed tissue and neuroimmune interactions. This has been demonstrated for eosinophils by NGF being essential for tissue infiltration since NGF-neutralizing antibodies have been shown to reduce the number of eosinophils in the dermis and subcutis in allergic dermatitis in an animal model [27]. NGF has further been suggested to promote the recruitment of eosinophils to nerves by upregulating the intracellular adhesion molecule-1 (ICAM-1) and vascular cell adhesion molecule-1 (VCAM-1), as observed in murine DRG neurons [28]. Immune cells can interact with these nerves by releasing NGF, as has been described for T cells [2], mast cells [6], macrophages, monocytes [9], and eosinophils [64] (Table 1; Figure 1). In neurons, NGF exhibits a similar trophic effect to BDNF through neuronal outgrowth and branching. This has been shown in allergic contact eczema, where increased nerve fiber length and higher NGF levels in the epidermis have been observed [29]. Furthermore, Peters et al. found a positive correlation between epidermal thickness and the number of NGF-positive nerve fibers in the skin of AD patients [65]. In non-lesional skin, itch was found to be correlated with the number of NGF-positive nerve fibers. Furthermore, a correlation between disease severity assessed by SCORAD (SCOring Atopic Dermatitis) and contacts of mast cells with nerve fibers in lesional skin was also observed [65]. NGFs’ influences on the level of NGF expression in mast cells were further investigated in a mouse model, where reduced NGF expression after anti-NGF treatment was observed [27]. Untreated, stressed mice with atopic dermatitis-like allergic dermatitis (AlD) also exhibited increased degranulation of mast cells, which is a sign of enhanced neurogenic inflammation [27]. This study also provided further evidence of a link between stress and NGF expression, as epithelial NGF, TrkA, and p75NTR were increased in stressed mice in comparison to mice without stress with AlD [27]. Interestingly, the precursor of the biologically active NGF, pro-NGF, was only detectable in stressed mice with AlD. This is of particular relevance as pro-NGF is known to signal via the sortilin receptor [66], potentially reversing the neuroprotective and pro-apoptotic effects [67]. The role of NGF in its own upregulation and that of its receptors under stress was demonstrated through decreased expression after treatment with NGF-neutralizing antibodies [27]. The impact of stress on AD has also been made apparent in another AlD mouse model in which mice were exposed to noise stress. It was observed that the number of eosinophils was significantly increased in lesions of stressed AlD mice compared to healthy mice or those without noise exposure [68]. This study also showed that epidermal thickness and the number of VCAM-positive blood vessels were elevated under stress [68], indicating that stress is able to contribute to inflammation in AD. Therefore, further investigations into the role of NGF in stress-induced exacerbation of AD are promising.

The importance of NGF in pruritic skin, such as in AD, also becomes apparent when taking a closer look at expression levels in healthy and AD patients. In eosinophils, NGF is localized in the central core of stable granules, which was revealed by investigating the peripheral blood of AD patients [30]. Expression of NGF is elevated in eosinophils from AD patients when compared to those of healthy patients [30]. However, levels of NGF expression differ not only in eosinophils but also in serum in health and disease [31]. In a previous study, we observed higher NGF levels in patients with the intrinsic and extrinsic types of AD than in healthy subjects, with no differences being noticeable between the two subtypes of AD, suggesting that NGF plays a role in inflammation in both subtypes. In contrast to the intrinsic type of AD, higher levels of total IgE and allergen-specific IgE are measurable in the extrinsic type. Additionally, patients presenting with the extrinsic type differ from those with the intrinsic type, with contrasting histories of AR and allergic asthma [31]. NGF levels in the stratum corneum might also function as a marker for AD, as correlations with pruritus and skin eruptions in AD have been described [69]. Furthermore, the stratum corneum of patients with psoriasis has higher NGF levels in pruritic lesions than in non-lesional skin, though levels are generally lower than those in AD [69]. Additionally, expression of the NGF receptor TrkA is increased in the epidermis and upper dermis [70], but also in human peripheral blood eosinophils in AD [39]. These findings suggest a pivotal role for NGF in AD in addition to its role in other atopic diseases.

### 3.2. NGF in Allergic Rhinitis and Asthma

As described for BDNF, NGF is also linked to AR, as expression of NGF in submucosal glands and nerve bundles in nasal mucosa was elevated 24 h after nasal provocation with allergens, whereas no change in NGF expression levels could be observed in healthy control subjects [14]. The allergen provocation also caused the number of NGF-positive nerve bundles in the nasal mucosa of AR patients to trend upward [14]. In AR, NGF expression in the nasal epithelium further exhibits a positive correlation with subepithelial protein gene product (PGP) 9.5 levels, indicating a possible impact of NGF on neuronal plasticity [71]. Another study also found significantly higher serum levels of NGF in patients with AR, allergic asthma, and urticaria-angioedema in comparison to control subjects [32]. For allergic asthma, higher NGF levels in BALF have also been reported and were found to be elevated even further after segmental allergen provocation [33]. Besides NGF, higher levels of SP have also been observed in the BALF of asthmatics, which is in accordance with the previously mentioned SP-inducing qualities of NGF. SP in turn is known to cause typical changes in the airways of patients with asthma, such as bronchoconstriction, vasodilatation, and increased mucus secretion. It is also known as a mitogen of smooth muscle cells, endothelial cells, epithelial cells, and fibroblasts, which suggests SP has a role in mediating the thickening of airways that can be observed in asthma [72]. The direct role of NGF in allergic asthma has been demonstrated in a mouse model of the disease. The numbers of eosinophils, neutrophils, macrophages, and lymphocytes in BALF after allergen provocation were found to be significantly increased in asthmatic mice by 3- to 8-fold in comparison to healthy control animals [34]. The role of NGF was confirmed through the application of anti-NGF treatment, which inhibited allergen-induced airway hyperreactivity and further reduced the number of eosinophils, neutrophils, and macrophages in BALF. Histological examinations also revealed a significant reduction of inflammatory infiltrates and bronchial damage after NGF antibody treatment, while serum IgE levels were also significantly reduced [34]. In the mediastinal lymph nodes, the percentage of Th1 and regulatory T cells increased, and the frequency of Th2 cells was found to be reduced after treatment with NGF antibodies [34]. These findings underline the importance of NGF in the balance of pro- and anti-inflammatory T cell responses in airway inflammation. However, hyperreactivity to allergens does not only influence the composition of immune cells but also changes receptor expression, as described in eosinophils. In BALF of patients with allergic asthma, upregulation of TrkA, but also TrkB, TrkC, and p75NTR in eosinophils, along with increased numbers of these immune cells, has been observed after allergen provocation [35]. Furthermore, BALF eosinophils also exhibit higher activation levels as measured by CD69 expression after stimulation with NGF, BDNF, NT-3, and NT-4/5. Stimulation of BALF eosinophils with NGF further identified the neurotrophin as an apoptosis-inhibiting factor [35]. Together, the findings of increased local NGF and receptor expression levels, the involvement of SP, and the tendency of NGF to induce Th2 inflammation indicate an important role for the neurotrophin not only in skin diseases such as AD but also in AR and allergic asthma.

### 3.3. NGF and Allergic Rhinoconjunctivitis

Another disease that atopic patients often suffer from is ARC. Early evidence of NGF being involved in eye diseases is provided by a study that observed higher NGF plasma levels and a correlation with the number of mast cells in the conjunctiva of patients with vernal keratoconjunctivitis [73]. Moreover, NGF levels in tears have been observed to be elevated in patients with ARC after allergen provocation [36]. Interestingly, patients with ARC who also presented with asthma showed even higher levels of NGF than patients with solely ARC. Differences in NGF levels were also observed between the perennial and seasonal types of ARC. Patients with perennial ARC showed more highly elevated NGF levels in tears after allergen provocation than patients with seasonal ARC, although no differences were observable without allergen provocation [36]. Therefore, the presence of subclinical allergic inflammation in patients with consistent allergen exposure was proposed. Furthermore, not only the neurotrophin but also the low-affinity receptor p75NTR is upregulated in the conjunctival epithelium of patients with ARC when compared to healthy controls. Since TrkA expression was not increased, this might indicate that NGF acts primarily via p75NTR in ARC [36]. Regarding the nerve-modulating effect of neurotrophins, it was shown that S1P upregulates NGF, which promotes corneal nerve repair in a mouse model [74]. This finding might suggest that NGF also plays a role in modulating nerves in the conjunctiva in ARC. This raises the hypothesis of sensitization to exogenous triggers. In summary, to date, NGF is the only neurotrophin that is known to be involved in ARC. These findings, however, indicate that it might be worth it to also investigate the role of the other neurotrophins in ARC, as their effects in other allergic diseases have been demonstrated to varying extents.

In conclusion, a wide variety of studies have demonstrated that aside from BDNF, NGF is the best-characterized neurotrophin in AD, AR, allergic asthma, and even ARC. The cytokine release-inducing and functional qualities in leukocytes, the effects on neuronal outgrowth, and higher levels of NGF in serum and local fluids suggest a pivotal role for the neurotrophin in these allergic diseases.

## 4. NT-3 and NT-4/5

The neurotrophins NT-3 and NT-4/5 are thus far not as well characterized as BDNF and NGF. NT-3 is about 50% homologous to NGF, BDNF, and NT-4/5 [75], while having its own exclusive receptor, TrkC [1]. NT-3 is released by keratinocytes, melanocytes, mast cells, eosinophils [37], monocytes [38], and T cells [4] (Table 1, Figure 1). Besides mast cells, all these cells are not only a source of but also responsive to NT-3 [37,38,76]. It has been reported that NT-3 leads to increased NGF secretion from keratinocytes, whereas NGF in turn enhances the release of NT-3 [37]. Among T cells, NT-3 is expressed in all CD4^+^ T cells, whereas its receptor TrkC is only expressed in Th2 cells, as demonstrated in mice [76]. Th2 cells are especially involved in allergic inflammation, for example in AD [61]. They respond to NT-3 with an enhanced release of IL-4 [76], which in turn elevates levels of other AD-related cytokines. In eosinophils, TrkC, as well as the low-affinity receptor p75NTR, is upregulated in AD patients compared to healthy controls. Stimulation of eosinophils from AD and AR patients with NT-3 has been shown to inhibit apoptosis significantly. NT-3 and NT-4/5 have also been shown to induce chemotaxis in peripheral blood eosinophils from AD patients [39]. Eosinophils further respond to NT-3 stimulation by significantly increasing their levels of eosinophil peroxidase [24]. Another indication of NT-3 having an important role in AD is that the mast cells of AD patients have a higher abundance of the neurotrophin than the mast cells of healthy subjects [40]. Initial data indicate that NT-3 might also play a role in AR since NT-3 levels in the nasal tissue of patients with AR are elevated compared to healthy subjects. However, NT-3 serum levels are not increased in AR patients, which suggests a locally confined release in nasal tissue [41]. NT-3 also might play a role in allergic asthma as levels in BALF of asthma patients after allergen provocation have been found to be elevated, which has previously also been described for NGF and BDNF [17]. NT-3 also promotes the survival of immune cells in BALF, as shown for eosinophils [35]. Another study investigated differences in NT-3 and NT-4/5 serum levels in asthmatic children [42]. The data showed a correlation between the serum levels of these neurotrophins and symptom severity. Children with moderate and severe asthma exhibited significantly higher NT-3 and NT-4/5 levels in the peripheral blood than those with mild asthma. However, differences between children with allergic asthma and those without an atopic background were not observed [42]. The neurotrophin NT-4/5 has been identified as being released by keratinocytes, melanocytes, macrophages [11], and mast cells [37] (Table 1, Figure 1). For this neurotrophin, increased expression in human epidermal keratinocytes from AD patients has also been demonstrated [43]. NT-4/5 has been shown to inhibit apoptosis in eosinophils from AR patients [39]. Furthermore, increased survival of BALF eosinophils in patients with allergic asthma after NT-4/5 stimulation has also been observed [35]. While the studies on NT-3 indicate an impact on inflammation in atopic diseases such as AD, AR, and allergic asthma, the role of NT-4/5 still needs to be determined. However, preliminary data on this neurotrophin indicate that it contributes to allergic diseases as well.

## 5. Neuronal Effectors

Specific neuronal populations differentially depend on neurotrophin signaling [77], such as NT-3-dependent touch-sensitive myelinated fibers and Merkel cells [78] and unmyelinated nociceptive neurons depending on BDNF and NGF [79], but also sympathetic efferent neurons critically depending on NGF. Beyond their developmental role, neurotrophins continuously modulate the neuronal excitability of adult neurons [75].

In human skin, NGF sensitizes nociceptors for several weeks [80], even leading to low-level ongoing pain when combined with local inflammation [81], and increases both cowhage-induced itch [82] and pruritogen-induced pain [83]. Moreover, NGF upregulates BDNF expression in primary afferent neurons [84], which, upon release in the spinal cord, further increases sensitization. Thereby, neuroimmune interactions can facilitate key allergic symptoms related to neuronal activation, such as itch and pain [85]. Based on major differences between nociceptor populations of rodents and humans [86], the translation of non-histaminergic itch might be problematic, but at least the key pruriceptive trkA-positive population of somatostatin- and natriuretic peptide B (nppb)-positive neurons that also express receptors for histamine (H1), IL-31, and leukotriene (LTC4) appear to be conserved between the species [87].

In asthma, neurons of the autonomous nervous system join spinal nociceptive fibers to innervate the lung: in particular, TrkB-positive parasympathetic vagal fibers and NGF-dependent sympathetic neurons [88]. While vagal and spinal nociceptive afferents provide the afferent information for sneezing and coughing, cholinergic parasympathetic fibers are crucial for tearing, rhinorrhea, and airway secretion, but also for the vasodilation that induces nasal congestion and ocular irritation. In contrast, adrenergic sympathetic efferents improve these symptoms via vasoconstriction and bronchodilation. It is important to note that neurotrophins are not the only mediators involved in the neuroimmune interaction of spinal nociceptors [89] and autonomic fibers [90]. However, neurotrophins play an important role in increasing the responsiveness of parasympathetic fibers [91] and thereby modulating allergic symptoms of the lower airways [92,93].

## 6. Conclusions

In conclusion, all neurotrophins have been observed to be expressed at higher levels in AD, AR, allergic asthma, or ARC than in healthy controls, either locally in inflamed tissue and immune cells or systemically. This underlines their pivotal role in these common atopic diseases. Particularly, BDNF and NGF are well characterized and linked to pruritic skin inflammation. This is demonstrated by neuroimmune interactions leading to outgrowth, branching, and higher density of nerves in affected skin and the ability to induce the release of cytokines associated with AD from immune cells such as eosinophils, basophils, and Th2 cells. Consequently, BDNF and NGF acting as mediators between neurons and eosinophils in AD is a promising aspect for improving our understanding of pruritus. The findings concerning NGF in AR, allergic asthma, and also ARC validate that these neurotrophins are worth putting into the spotlight of investigation. The functions of NT-3 and NT-4/5 in atopic diseases deserve more attention, as first insights suggest that these neurotrophins also play an important role in AD, AR, and allergic asthma.

## Figures and Tables

**Table 1:** Functions and upregulation of neurotrophins in human immune cells.

Neurotrophins	Expressing Immune Cells in Humans	Functions and Upregulation in Allergic Diseases	Refs.
BDNF	Eosinophils, T cells, B cells, macrophages, monocytes, mast cells, platelets	Atopic dermatitis elevated in plasma, serum, neurons, and eosinophils released by eosinophils in close proximity to peripheral nerves causes outgrowth and branching of nerve fibers inhibits apoptosis and induces chemotactic migration of eosinophils Allergic rhinitis elevated in serum and nasal mucosa after allergen provocation Allergic asthma increased serum levels are controversial levels in platelets correlate with bronchial hyperresponsiveness higher levels in BALF after allergen provocation impact on the contractility of smooth muscle cells in the airways by regulation of calcium release from SR	[3,7,11,12,13,14,15,16,17,18]
NGF	Eosinophils, T cells, macrophages, monocytes, mast cells	Skin inflammation inhibits apoptosis and increases the expression of IL-4 in eosinophils induces secretion of IL-13 in basophils modulates basophil functions by enhancing histamine and leukotriene C4 (LTC4) release upregulates SP and CGRP induces mast cell degranulation promotes recruitment of eosinophils to nerves causes outgrowth and branching of nerve fibers elevated in serum and eosinophils in AD and correlated with disease severity Allergic rhinitis increased in submucosal glands and nerve bundles after allergen provocation higher serum levels Allergic asthma elevated in serum and BALF after allergen provocation increases number of immune cells in BALF after allergen exposure activates BALF eosinophils Allergic rhinoconjunctivitis elevated in tears after allergen provocation	[2,14,19,20,21,22,23,24,25,26,27,28,29,30,31,32,33,34,35,36]
NT-3	Eosinophils, T cells, monocytes, mast cells	induces chemotaxis of peripheral blood eosinophils in AD elevated in mast cells in AD and in nasal tissue in AR higher levels in BALF after allergen provocation in asthma increased levels in the peripheral blood of asthmatic children	[4,17,37,38,39,40,41,42]
NT-4/5	Macrophages, mast cells	induces chemotaxis of peripheral blood eosinophils in AD increased levels in the peripheral blood of asthmatic children higher expression in epidermal keratinocytes in AD inhibits apoptosis of eosinophils in AR and asthma	[11,35,37,39,42,43]

## Data Availability

No new data were created or analyzed in this study. Data sharing is not applicable to this article.
